# Effectiveness of Social Media Approaches to Recruiting Young Adult Cigarillo Smokers: Cross-Sectional Study

**DOI:** 10.2196/12619

**Published:** 2020-07-22

**Authors:** David Cavallo, Rock Lim, Karen Ishler, Maria Pagano, Rachel Perovsek, Elizabeth Albert, Sarah Koopman Gonzalez, Erika Trapl, Susan Flocke

**Affiliations:** 1 Department of Nutrition School of Medicine Case Western Reserve University Cleveland, OH United States; 2 Center for Community Health Integration School of Medicine Case Western Reserve University Cleveland, OH United States; 3 Division of Child Psychiatry Department of Psychiatry Case Western Reserve University Cleveland, OH United States; 4 Prevention Research Center for Healthy Neighborhoods Departments of Population and Quantitative Health Sciences Case Western Reserve University Cleveland, OH United States; 5 Department of Family Medicine Oregon Health & Science University Portland, OR United States

**Keywords:** adolescent, young adult, tobacco products, social media, research subject recruitment

## Abstract

**Background:**

The prevalence of social media use among youth and young adults suggests it is an appropriate platform for study recruitment from this population. Previous studies have examined the use of social media for recruitment, but few have compared platforms, and none, to our knowledge, have attempted to recruit cigarillo users.

**Objective:**

The purpose of this study was to examine the effectiveness of different social media platforms and advertisement images for recruiting cigarillo users aged 14-28 years to complete a cigarillo use survey.

**Methods:**

We obtained objective data for advertisement impressions for a 39-week social media recruitment campaign. Advertisements were targeted to users based on their age, geography, and interests. Effectiveness was defined as the percentage of approved surveys per advertising impression. Chi-square tests were performed to compare the effectiveness of different advertisement images and platforms.

**Results:**

Valid survey completers (n=1089) were predominately older (25-28 years old, n=839, 77%). Of the 1089 survey completers, 568 (52%) identified as male, 335 (31%) as African American, and 196 (18%) as Hispanic. Advertisements delivered via Facebook/Instagram were more effective than Twitter; 311/1,027,738 (0.03%) vs 661/2,998,715 (0.02%); *χ*^2^_1_=21.45, N=4,026,453); *P*<.001. Across platforms, ads featuring exclusively an image of cigarillos were more effective (397/682,994, 0.06%) than ads with images of individuals smoking (254/1,308,675, 0.02%), individuals not smoking (239/1,393,134, .02%), and groups not smoking (82/641,650, 0.01%); *χ*^2^_3_133.73, N=4,026,453; *P*<.001.

**Conclusions:**

The campaign was effective in recruiting a diverse sample representative of relevant racial/ethnic categories. Advertisements on Facebook were more effective than Twitter. Advertisements that featured an image of a cigarillo were consistently the most effective and should be considered by others recruiting cigarillo users via social media.

## Introduction

Recent data on smoking prevalence indicates that a substantial proportion of youth in the US smoke cigar products, including cigarillos [1]. Like other tobacco products, cigarillo use increases the risk of cancer, cardiovascular disease, and mortality, and some early evidence suggests that cigarillos may be more harmful to organ systems than cigarettes [2]. Cigarillo use is also concentrated among young, urban, minority populations that have traditionally experienced health disparities related to tobacco use and may be more vulnerable to the long-term harmful effects of smoking [3]. Considering the public health risk presented by cigarillo use, understanding patterns of use and the addictive properties of these products via survey research could serve as the first step in reversing current trends in their adoption and use by youth and young adults.

Recruiting cigarillo users for survey research presents unique challenges using traditional methods. Youth and young adults are less likely to respond to survey recruitment via telephone and change their residency often, making mail-based recruitment less effective [4]. Youth and young adults may also feel more comfortable completing surveys addressing behaviors such as tobacco use, which may be illegal or stigmatized among these participants, online rather than in a school setting or other public venue [5]. Web-based recruitment may, therefore, be more appropriate given the increasing comfort and amount of time spent with digital platforms in this population and the relative anonymity of accessing and completing surveys online [6].

Web-based recruitment using social media may be particularly appropriate in reaching youth and young adult cigarillo users [7]. In addition to being delivered on the web, social media reaches a diverse group of users, including significant proportions of low-socioeconomic status, minority populations, especially among younger age groups [8]. Advertising on social media also allows for the specific targeting of these groups using demographic characteristics as well as specific behaviors associated with tobacco use [9]. There is also a seamless user recruitment experience as social media advertisements link participants directly to online screeners and surveys.

A growing body of research has examined the use of social media advertisements for tobacco research recruitment. Studies have found social media advertising recruitment to be more efficient for recruiting smokers when compared to specialized mailings [10], research databases, course-based recruitment [11], and online panels [12]. Conversely, school-based referrals, bus ads, participant referrals, and fliers were found to be more cost-effective than social media when recruiting adolescent smokers [13]. Other studies that did not compare social media to alternative recruitment methods describe social media as effective in recruiting young adult smokers for survey and intervention research [14,15].

Most studies examining social media advertising recruitment have used Facebook [7]. The few direct comparisons of Twitter and Facebook for study recruitment have yielded mixed results, primarily used interpersonal modes of social media recruitment vs paid advertising, and assessed effectiveness with nonmonetary outcomes [16,17]. Given the significant risk of cigarillo use among youth and young adults, the growing evidence of the efficacy of social media in recruiting young adult smokers, and a lack of evidence supporting effective social media advertising strategies, the purpose of the current study was to examine the relative effectiveness of using Facebook, Instagram, and Twitter to recruit cigarillo users to complete an online nicotine dependence survey and provide guidance for future efforts using social media to study cigarillo use behavior.

## Methods

### Recruitment

**Figure 1:**
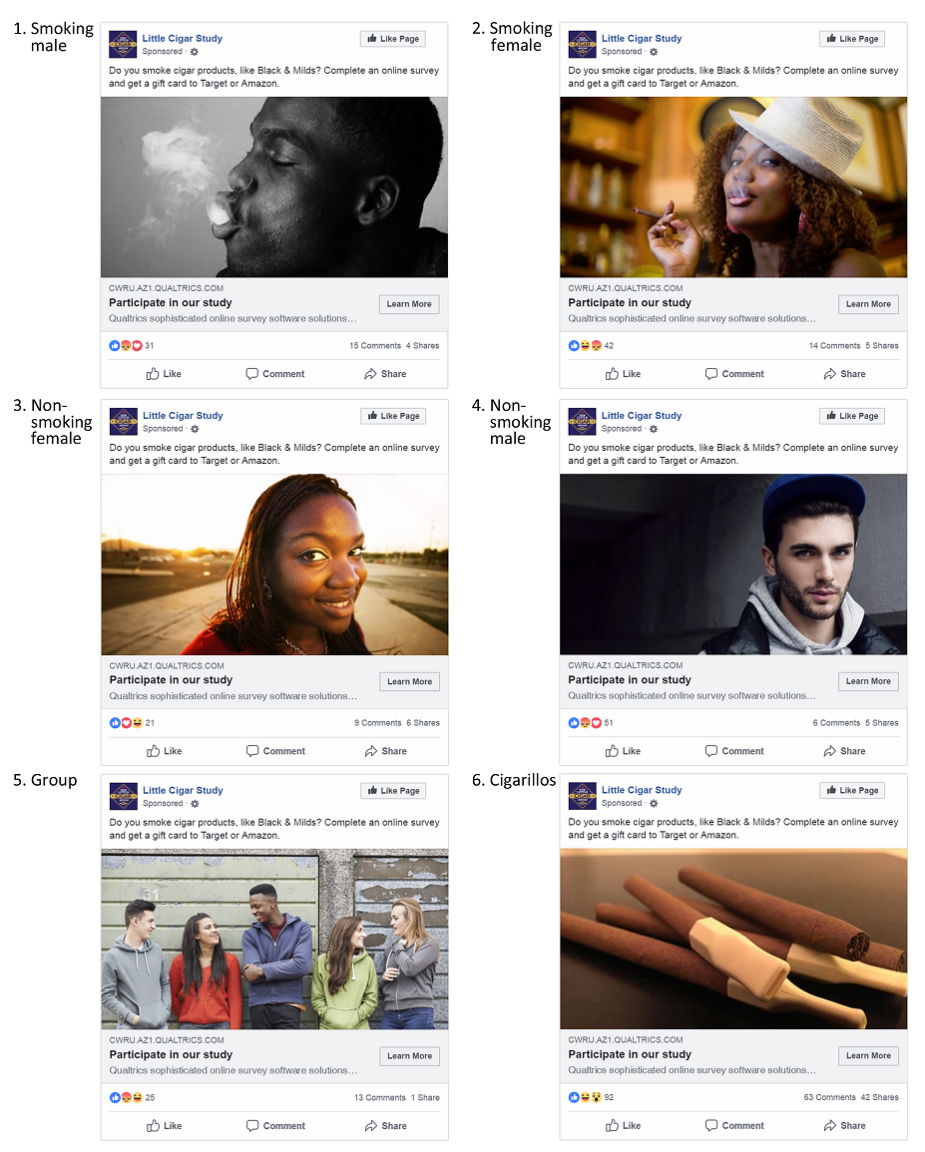
Facebook advertisement examples.

Participants between 14 and 28 years old who smoked at least two cigarillos per week were considered eligible. Participants also had to read and understand English and have access to an internet-connected device. Study procedures were approved by the Case Western University Institutional Review Board, and participants provided informed consent. Our study did not collect individually identifiable data from social media platforms, reducing risks related to social media data privacy and anonymity recently raised in the research community [18]. Six social media advertisements were developed to run simultaneously on Facebook, Instagram, and Twitter platforms. Because advertisements were run together on a combined Facebook/Instagram campaign with the same URL, we were unable to separate the results for Facebook and Instagram at the survey completion level. We will refer to these combined platforms as “Facebook” when describing analyses for survey initiation and valid completion. To partially address this limitation, we analyzed advertising effectiveness between Facebook and Instagram at the level of advertisement clicks, which is available from the advertising management platform. Each advertisement contained identical wording that encouraged viewers to participate and featured a direct link to a brief eligibility screener administered via Qualtrics (Figure 1).

Advertisements featured planned images representing characteristics we intended to evaluate for advertising effectiveness (a cigar product, a smoking individual, a group of people, and gender). Advertisements were targeted to geographic areas reporting a high prevalence of cigarillo use (Cuyahoga County, OH; Baltimore, MD; Broward County, FL; Detroit, MI; DeKalb County, GA; Houston, TX; Philadelphia, PA; Washington, DC, Duval County, FL; and Fort Worth, TX) [1]. Advertisements were delivered daily for approximately 4 weeks in each location over 39 weeks beginning in June of 2017 and ending in March 2018. Advertisements were targeted using the age range most similar to our inclusion criteria that were an available option. We targeted Facebook advertisements to users 14-28 years old during the entire campaign and Twitter advertisements to users 13-34 years old, starting in November, once that feature became available. Advertisements were also targeted on relevant keywords such as “cigarillo” and “blunt” that were considered relevant to the target population. We did not encourage the sharing of social media advertisements through advertising messages or any other mechanism.

A budget of $240/day was set, and advertisements were scheduled to run with identical budgets so that advertisements would be delivered with similar frequency by the advertising platform algorithm. This method was essential to allow a fair comparison of advertisements as less effective advertisements may run for a brief time when using social media advertising optimization algorithms. We used the cost/click method of payment traditionally used by advertisers wishing to direct users to a website whereby costs are based on an online auction for individual clicks on the advertisement.

### Survey

Individuals who completed the screener and met the eligibility criteria were invited to take the survey. Invitations were delivered via a brief email or text message, based on participant preference, and included a link to a survey administered via Qualtrics. Reminders were sent via the preferred method at 2, 5, and 8 days after initially sending the survey link. Respondents who submitted valid surveys were remunerated with a $15 gift card.

The survey included questions assessing several dimensions relevant to cigarillo consumption, including demographic information, nicotine dependence, brand preference, group smoking behavior, psychosocial constructs, and the sequence of product initiation. Several sections of the survey used branching logic such that for each tobacco product endorsed, additional questions were assessed. Therefore, the number of survey items varied based on participants’ reported product use.

Because nomenclature for tobacco products varies across audiences and geographically [19], several features were incorporated into the questions about tobacco products to ensure that items about tobacco products would be consistently understandable across participants with varying demographic and geographic characteristics. The questions referring to tobacco products used: the name of the product type (eg, “tipped cigarillo”), a familiar product brand (eg, “Black & Mild”), and a photograph of the product. Instructions and survey items were written at a 6^th^-grade literacy level. On average, the survey took about 30 minutes to complete.

#### Strategies to Ensure the Quality of the Survey Data

Multiple strategies were used to ensure the quality of online survey data, which can be adversely affected by respondents who falsely complete surveys for financial gain [20]. First, for the screener survey, we analyzed contact information items (email address and phone number) for validity and consistency and to identify possible repeat respondents [21]. Second, the screener survey included an open-answer item asking for a brief description of how participants got started smoking little cigars or cigarillos. This item required a multiple word response and was used to screen out responses generated by computer programs. This question was manually evaluated before a link to the survey was sent to the eligible participant. Third, a question about how the participant heard about the survey was used to ensure that participants came across the survey legitimately. If they claimed to hear about the survey through a channel that was not used by us, their responses were flagged and examined more carefully.

Completed surveys were manually assessed by study staff for validity before analysis. Validation was performed through the use of “trap” or “red herring” questions [22], checking for surveys completed abnormally fast or with poor response consistency [23], checking for unusually repetitive responses (“straight-lining”) [24], and checking response sets with similar email addresses or IP addresses. Surveys that exhibited these characteristics were reviewed more carefully. Suspected repeat responders (based on emails or IP addresses) were rejected if there was sufficient evidence (based on the reviewer’s judgment) to do so. For example, if respondents with the same IP address gave similar answers, the surveys were completed one immediately after the other, or the respondents had emails sufficiently similar to suggest they were, in fact, the same person. Suspected satisficing cases were reviewed and either outright rejected or placed in a questionable category for further review. Multiple members of the study staff then reviewed these cases to make a final determination to accept or reject the survey.

### Measures

Data on advertising effectiveness were obtained objectively from the Facebook/Instagram and Twitter advertising analytics platforms. Data included advertisement impressions (number of individuals to whom the advertisement was delivered), cost, number of times an advertisement was clicked, and number of times an advertisement was shared on the social media platform. The advertisement that generated each survey was identified by linking the response to a unique URL. Survey data included demographics and metadata related to survey and screener completion.

### Statistical Analysis

Statistical analyses were conducted with SAS (Version 9.2) and SPSS (Version 25). Descriptive statistics were used to assess participant characteristics and sharing rates. Advertising effectiveness by platform and content is expressed as ratios. These included the number of screeners started per impression, the number of valid surveys per impression, and the cost per started screener and valid survey. We chose started screeners and valid surveys as the indicators of recruitment effectiveness except for the analyses of Facebook vs Instagram, where clicks were used as a metric of effectiveness. We also calculated the time in hours from when a participant started a screener to when they completed a survey. Differences between advertisement effectiveness were examined using Pearson chi-square tests. Analysis of which participant characteristics predicted recruitment advertising platform was performed using logistic regression models. Differences in survey time to completion were examined using *t* tests.

## Results

**Figure 2:**
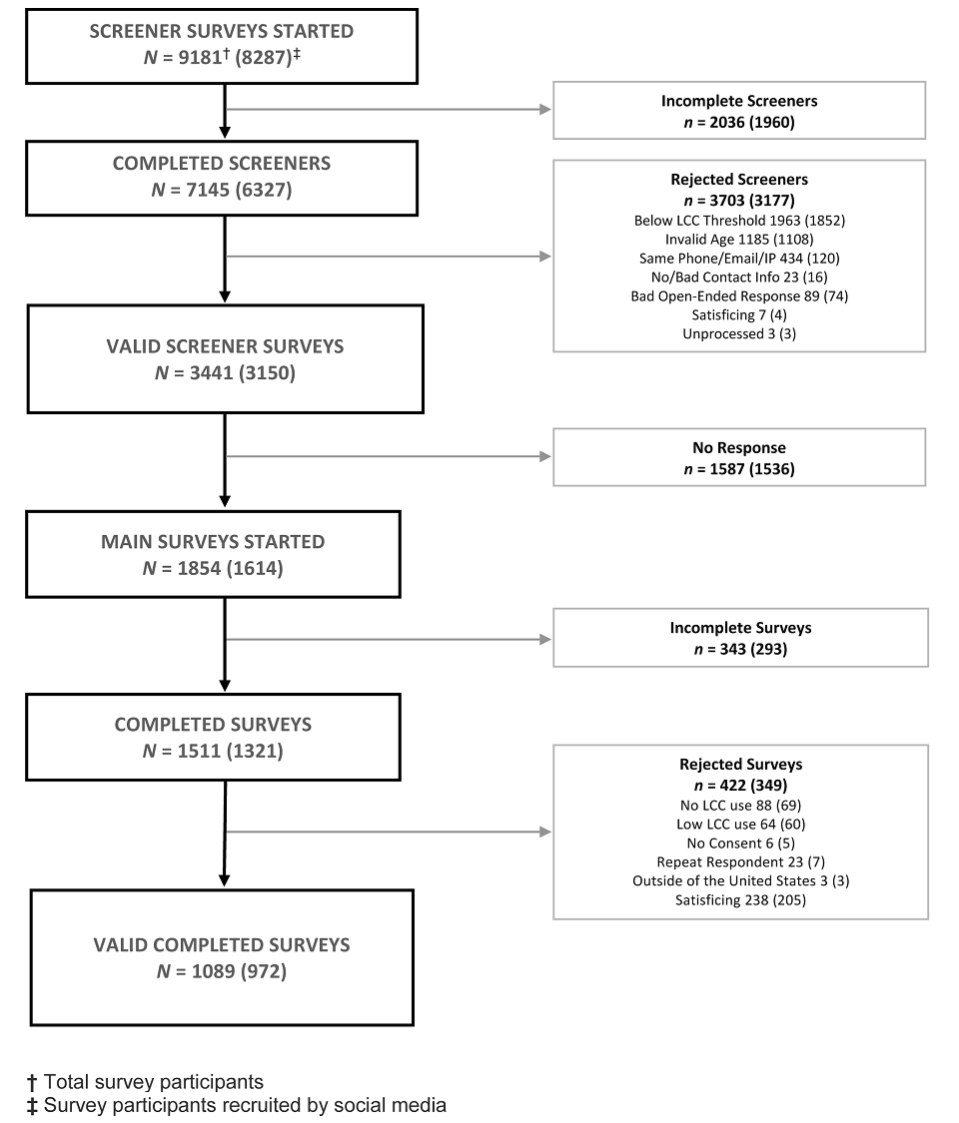
Screener to survey completion flowchart.

The combined social media advertising campaign generated 4,026,453 impressions, and 8287 started screeners. Of 6327 completed screeners, 3150 (50%) were identified as eligible and were sent a link to the survey (Figure 2). Slightly more than half of the eligible participants started the survey (1614/3150, 51%), and 1321 of those completed it. Of the completed surveys, 349 were rejected due to a low level of cigarillo use, satisficing, or not meeting other review criteria. Table 1 shows the demographic characteristics of the total valid survey completers (n=1089) and valid survey completers who were recruited using social media (n=972). We found no significant differences in gender, age, race/ethnicity, and education between those who were recruited via Twitter or Facebook and subjects recruited by other methods (eg, palm cards, referral by another participant, n=117). Of note, 509 (47%) of the total sample were female, 839 (77%) were aged 25-28, 335 (31%) were African American, and 196 (18%) were Hispanic. The median number of cigarillos per day for all valid survey completers was 0.93. In addition, 585 (53.7%) also smoked cigarettes, and 866 (79.5%) reported using at least one other combustible tobacco product in the past 30 days.

Tables 2-3 describe advertisement effectiveness and cost comparisons. There was no significant difference between the percent of started screeners by impressions between Facebook and Twitter. Advertisements featuring a female image were more successful in generating started screeners than those with male images, and advertisements with a cigarillo image were more effective than those without. Facebook generated a greater percentage of valid surveys per impression than Twitter. There was no significant difference by the gender of the advertising image, and advertisements with a cigarillo image again were markedly more effective. Facebook was less cost-effective for generating started screeners but more cost-effective for producing valid surveys. Facebook was significantly more effective in generating clicks on the study advertisement per impression than Instagram (n=8507, 1.76% vs n=1198, 1.16%, *χ*^2^_1_=188.24, *P*<.001, N=585,648). Instagram was also more expensive than Facebook per click obtained ($1.32 US vs $0.81 US). Facebook and Twitter significantly differed in the interval between respondents starting a screener and completing a survey (mean 66.63 h, SD 69.60 vs mean 80.03 h, SD 79.14; *t*=2.55_967_, *P*=.01). Across platforms, advertisements were shared 310 times (79 on Facebook, 231 on Twitter).

Analysis of the predictors of social media recruitment platform is detailed in Table 4.

Gender and race/ethnicity of survey completers did not predict through which social media platform a participant was recruited. The odds of being recruited by Facebook (n=310) versus Twitter (n=659) were 1.5-fold higher for those aged ≥21 years when compared to adolescents. Those recruited via Facebook were also more likely to report lower levels of education.

**Table 1:** Demographic characteristics and tobacco use of valid survey completers.

Characteristic		Total valid survey completers (N=1089), n (%)	Valid survey completers recruited via social media (N=972), n (%)
**Age**			
	14-18	19 (1.7)	15 (1.54)
	19-24	231 (21.2)	202 (20.78)
	25-28	839 (77.0)	755 (77.67)
**Gender**			
	Female	509 (46.7)	448 (46.1)
	Male	568 (52.2)	514 (52.9)
	Other	12 (1.1)	10 (1.0)
**Race/ethnicity**			
	Black or African American	335 (30.8)	309 (31.9)
	Hispanic	196 (18.0)	170 (17.5)
	White	436 (40.1)	385 (39.7)
	Other	120 (11.0)	106 (10.9)
**Education**			
	<GED or high school	109 (10.0)	98 (10.1)
	GED or high school	235 (21.6)	199 (20.5)
	Some college	432 (39.7)	396 (40.8)
	Associate	93 (8.5)	83 (8.6)
	BA+	219 (20.1)	195 (20.1)
**Tobacco use**			
	Number of cigarillos smoked/day (median)	0.93	0.90
	Cigarette smoker	585 (53.7)	520 (53.5)
	Smoked at least one other combustible tobacco product	866 (79.5)	772 (79.4)

**Table 2:** Advertising effectiveness by platform, image type, and cost (N=4,026,453).

Advertisement type			Impressions, n (%)	Started screeners	%	Chi-square (*df*)	*P* value
All			4,026,453 (100)	8,287			
**Platform**							
		Twitter	2,998,715 (74)	6,203	.21	0.62 (1)	.44
		Facebook	1,027,738 (26)	2,084	.20		
**Image gender**							
	Female		1,303,549 (32)	2,249	.17	15.55 (1)	<.001
	Male		1,398,260 (35)	2,142	.15		
**Image type**							
	Cigarillo		682,994 (17)	3,196	.47	941.75 (3)	<.001
	Smoking		1,308,675 (32)	2,174	.17		
	Nonsmoking		1,393,134 (35)	2,217	.16		
	Group		641,650 (16)	700	.11		

**Table 3:** Advertising effectiveness continued.

Advertisement type			Valid surveys		%		Chi-square (*df*)		*P* value		Cost/started screener		Cost/valid survey
All			972								$4.73		$40.34
**Platform**													
	Twitter	661		0.02		21.45 (1)		<.001		$4.63		$43.41	
	Facebook	311		0.03						$5.05		$33.82	
**Image gender**													
	Female	246		0.02		0.54 (1)		.38		$5.65		$51.69	
	Male	247		0.02						$6.17		$53.54	
**Image type**													
	Cigarillo	397		0.06		133.73 (3)		<.001		$2.19		$17.62	
	Smoking	254		0.02						$5.99		$51.26	
	Nonsmoking	239		0.02						$5.83		$54.06	
	Group	82		0.01						$8.97		$76.54	

**Table 4:** Odds of recruitment via Facebook vs Twitter (N=969).

Demographic			Coefficient		SE		*P* value		OR^a^ (95% CI)
Male gender (vs female and other)			–0.282		.145		.06		0.755 (0.568-1.002)
Age 21-28 (vs 14-20)			0.372		.180		.04		1.451 (1.019-2.065)
**Race/ethnicity**									
	Black or African American	–0.247		.176		.16		0.781 (0.553-1.104)	
	Hispanic	0.104		.198		.60		1.110 (0.752-1.637)	
	Other	0.113		.239		.64		1.120 (0.701-1.788)	
**Education**									
	<GED or high school	1.422		.304		<.001		4.147 (2.286-7.521)	
	GED or high school	1.447		.250		<.001		4.250 (2.603-6.939)	
	Some college	0.983		.228		<.001		2.672 (1.710-4.173)	
	Associate’s degree	1.019		.306		<.001		2.770 (1.519-5.050)	

^a^OR: odds ratio.

## Discussion

This study was successful in recruiting a large sample of youth and young adult cigarillo users, supporting the use of social media-based advertising to reach this group, which traditionally presents a challenge to survey and study recruitment efforts. The recruited sample was diverse and representative of individuals most likely to use cigarillos, with over a third of our sample consisting of African Americans, a demographic group with the highest reported cigarillo use among adults [3].

Advertisements on Facebook were more effective than Twitter in producing valid surveys. Facebook was also a more cost-effective recruitment method. Differences observed in the cost per approved survey (approximately $10 US) could represent a significant increase in the overall costs of recruitment in extensive studies. A limited number of previous studies have compared Facebook and Twitter research recruitment. A feasibility study examining the use of QR codes, Facebook, and Twitter for recruiting adolescents to take a health-related survey found Twitter to be marginally more cost-effective than Facebook [16]. That study, however, used student seeds to deliver Twitter recruitment as opposed to advertising. In another study that recruited focus groups about vaccination, Facebook was found to be more efficient than Twitter when comparing staff time spent per questionnaire received, but this study also used individual Facebook and Twitter accounts, not advertisements [17].

Our cost per valid survey of $40 US falls within a broad range of previous findings related to the cost-effectiveness of social media recruitment of tobacco users. Ramo et al reported substantially lower Facebook costs of $8.80 US per eligible, consented participant in a tobacco cessation intervention and $4.28 per completed survey in a separate study, both targeting young adults [14,15]. Other recent studies recruiting tobacco-related survey participants reported a cost per completed survey of $1.86 US (Facebook) [11], $21.73 US (Twitter) [12], and a cost per enrolled smoker for randomized controlled trials ranging from $41 to $62 US (Facebook) [25,26]. Many factors affect social media advertising costs, including seasonality, industry type, and the specificity of the audience being targeted [27]. It might be the case that a more specific audience (eg, individuals who smoke cigarillos) reduces the cost-effectiveness of the advertisements. In addition, social media advertising costs have risen in recent years as more advertisers use the platform and as a result of recent changes to the advertising algorithm, which may account for some of the increase from previous studies [28]. When comparing social media costs in the current study to other modalities for recruiting tobacco users, Facebook and Twitter advertisements fall in the middle of the cost-effectiveness range. For instance, Brodar et al reported a cost per enrolled smoker in an experimental trial ranging from $7 US using Craigslist to $375 US for newspaper advertisements [25].

Our results also demonstrate that differences in effectiveness are a function of the metric used. Twitter was as effective in generating started screeners as Facebook but was less effective in generating approved surveys. Investigators should use the measure of effectiveness most closely related to their recruitment goal as opposed to more distal measures such as advertising clicks that are more accessible via social media advertising platforms. Participants recruited via Facebook also completed the process of producing a valid survey more rapidly than those recruited via Twitter. For large studies or those on a short timeline, this measure of performance should be considered to ensure that the study goals are met. We observed minimal naturally occurring sharing of advertisements across platforms when compared to the reach of paid advertising. The viral nature of social media platforms, however, lends itself to this mode of recruitment, and researchers are beginning to use this strategy for a variety of recruitment and communications tasks [29,30]. Given the fact that sharing is a cost-free method of disseminating social media advertisements, future research should test methods for increasing the effectiveness of viral social media survey recruitment, such as nonmonetary rewards or small monetary incentives.

Regardless of the platform, the inclusion of a cigarillo image was consistently the most effective advertisement design. A similar result was found in a comparison of Facebook advertisement images used to recruit young smokers, where the advertisement with an image of a cigarillo had the lowest cost per unique click of 4 images used [15]. This strategy was also used in another successful Twitter recruitment campaign where all advertisements featured a prominent image of a tobacco product [12]. The impact of the image may be the result of having a strong visual cue similar to existing cigarillo marketing or the simplicity of the image given the limited space available for social media advertisements.

This study has several strengths. We had a large sample of advertising impressions on which to base our analysis. We created individual URLs for each advertisement on each platform linked to individual participants’ screeners and surveys, allowing us to examine more relevant forms of effectiveness and assess demographic factors associated with social media platform advertising effectiveness. Limitations include our inability to examine Facebook and Instagram separately at the same level of effectiveness as our other analyses. The demographic profile of these social media platforms differs on several key demographic factors such as age and race, which may create differences in their effectiveness as suggested by our results comparing clicks per impression. In addition, over the past five years, Instagram use has consistently increased while Facebook has leveled off [31]. Understanding the effectiveness of Instagram should increase in importance if this trend continues. This study is not experimental, meaning that individuals were not randomized to the social media platform exposure. This limitation creates the possibility that participants may have been exposed to advertisements from Facebook and Twitter and responded only after being exposed to a specific dose. Additionally, the targeting features of social media platforms, such as geo-targeting, are inferred based on data collected in part through self-report, which may reduce their accuracy.

Social media platforms should be considered for tobacco-related research, especially in studies attempting to recruit hard-to-reach populations, such as youth, young adults, and minorities. This method is cost-effective when compared to other modalities and provides a convenient means of digital recruitment on a nationwide basis. Although in the current study, Facebook was a more cost-effective platform, given the fact that we observed some differences in the likelihood of recruitment platform between age and education levels and our inability to determine how many individuals in the sample use both, it may be that using multiple platforms remains the optimal recruitment strategy. Using multiple social media platforms may be less cost-effective, but by increasing the potential audience size, researchers may improve the ability to reach subgroups and recruit rapidly, if required.
